# Quantifying the heritability of testicular germ cell tumour using both population-based and genomic approaches

**DOI:** 10.1038/srep13889

**Published:** 2015-09-09

**Authors:** Kevin Litchfield, Hauke Thomsen, Jonathan S. Mitchell, Jan Sundquist, Richard S Houlston, Kari Hemminki, Clare Turnbull

**Affiliations:** 1 Division of Genetics and Epidemiology, The Institute of Cancer Research, London, SW3 6JB, UK; 2 German Cancer Research Center (DKFZ), Division of Molecular Genetic Epidemiology, Heidelberg, Germany; 3 Center for Primary Health Care Research, Lund University, Malmö, Sweden; 4 Stanford Prevention Research Center, Stanford University School of Medicine, Stanford, CA, USA; 5 William Harvey research Centre, Queen Mary University London, London

## Abstract

A sizable fraction of testicular germ cell tumour (TGCT) risk is expected to be explained by heritable factors. Recent genome-wide association studies (GWAS) have successfully identified a number of common SNPs associated with TGCT. It is however, unclear how much common variation there is left to be accounted for by other, yet to be identified, common SNPs and what contribution common genetic variation makes to the heritable risk of TGCT. We approached this question using two complimentary analytical techniques. We undertook a population-based analysis of the Swedish family-cancer database, through which we estimated that the heritability of TGCT at 48.9% (CI:47.2%–52.3%). We also applied Genome-Wide Complex Trait Analysis to 922 cases and 4,842 controls to estimate the heritability of TGCT. The heritability explained by known common risk SNPs identified by GWAS was 9.1%, whereas the heritability explained by all common SNPs was 37.4% (CI:27.6%–47.2%). These complementary findings indicate that the known TGCT SNPs only explain a small proportion of the heritability and many additional common SNPs remain to be identified. The data also suggests that a fraction of the heritability of TGCT is likely to be explained by other classes of genetic variation, such as rare disease-causing alleles.

Testicular germ cell tumour (TGCT) is the most common cancer in young men, with over 18,000 new cases of TGCT diagnosed annually in Europe^1^,^2^. Two main histological subtypes of TGCT are recognised—seminomas, which resemble undifferentiated primary germ cells and non-seminomas, which show differing degrees of differentiation. The incidence rate of TGCT has approximately doubled over the last 40 years in Western Europe^3^, which strongly implicates environmental or lifestyle factors as risk determinants. Molecular and clinical observations are consistent with the first oncogenic transformative step of the progenitor testicular germ cell occurring during fetal development^4^,^5^,^6^. However, despite extensive epidemiological study including maternal gestational exposures, to date no exogenous risk factors have been consistently associated with TGCT^7^. In contrast twin and family studies have provided robust evidence for inherited genetic susceptibility^8^,^9^. Direct evidence for inherited genetic susceptibility to TGCT has also come from recent genome-wide association studies (GWAS), which have so far identified 19 independent risk loci^10^,^11^,^12^,^13^,^14^,^15^,^16^,^17^,^18^

Given the importance of both environmental and genetic factors in the development of TGCT quantifying the contribution of heritable factors (*i.e.* the proportion of phenotypic variation due to genetic variance between individuals) is important in understanding the aetiological basis of this cancer. Despite the success of recent GWAS, the heritable nature of TGCT is poorly understood, both in terms of its magnitude and genetic architecture. Emergent statistical methods such as genome-wide complex trait analysis (GCTA) and phenotype correlation-genotype correlation (PCGC) regression allow the heritability ascribable to all common SNPs to be estimated from GWAS datasets^19^,^20^,^21^. These methodologies are complimentary to population based analyses, which quantify heritability from the clustering of disease within families.

Here we employ both methodologies to estimate the heritability of TGCT, by firstly performing an analysis of the Swedish population registry, comprising 15.7 million individuals and secondly conducting a GCTA analysis of a GWAS dataset of 6,000 individuals.

## Results

### Heritability estimate based on population data

**Figure 1:**
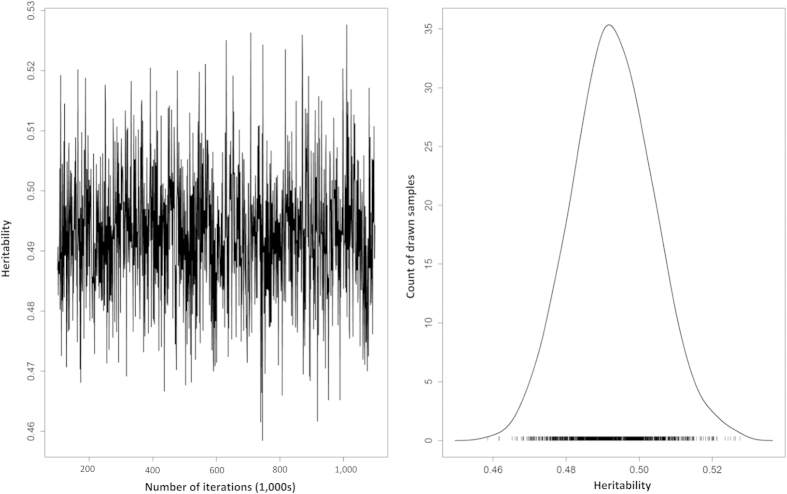
Trace and posterior density of population based heritability estimate.

Figure 1 shows a trace plot of the heritability values across the 1,000 sampled iterations. The trace shows the parameter space is evenly sampled, with good mixing, no biased trend and rapid convergence. The right side of Fig. 1 shows the posterior density of the heritability estimates and averaged across the 1,000 samples the posterior mean was 48.9% (95% confidence interval (CI): 47.2% – 52.3%). Heritability was also estimated for each histological sub-type, yielding values for seminoma and non-seminomas of 48.1% [95% CI: 43.4%–54.8%] and 49.6% [95% CI: 44.2%–55.1%] respectively. To assess the possible cohort effects of our estimates we calculated the heritability based on data for historical (1958–1992) and recent (1993–2012) time periods, however no significant difference in heritability was observed.

### Heritability estimates based on genomic data

After transforming the data to account for effective prevalence and ascertainment on the liability scale the heritability of TGCT explained by all autosome SNPs was 37.4% (95% confidence interval (CI): 27.6%–47.3%). The estimated heritability from PCGC regression was very similar—39.4% (95% CI: 20.9%–57.9%) suggesting that there was no calculation bias.

Sub-analyses were performed using GCTA, to investigate the underlying architecture of TGCT heritability. The first of these analyses assessed the relative contribution of individual chromosomes (Table 1), for which we observed a moderate correlation between heritability and chromosome length (Pearson’s correlation coefficient r = 0.56, *P* = 6.7 × 10^−3^). Chromosomes 3 and 1 were observed to contribute the most towards TGCT heritability, explaining 5.1% and 4.2% of phenotypic variance respectively, perhaps reflecting that in addition to the large size of these chromosomes, 4 of the 19 risk loci identified localised here. Chromosome 20 provided the third highest contribution to the heritability explaining 3.4%; somewhat intriguing as a risk locus has yet to be shown to localise to this short chromosome.

Following on from this we quantified the contribution from the 19 established TGCT risk loci to the overall variance (Table 2). Of note was the impact of rs995030 at 12q21 which was high, at just under 2%. Collectively all 19 loci accounted for 9.1% of the variance; translating to approximately one quarter of the total heritability (37.4%) expected to be explained by all SNPs. Finally, to explore the possibility that heritability for TGCT might be subtype dependent, a stratified analysis was conducted of seminoma (n = 385) and non-seminoma (n = 306); we did not consider patients with mixed or indeterminate histology. Surprisingly the results showed a higher heritability for seminomas 42.1% (95% CI: 21.1%–62.9%) as compared with non-seminoma 29.4% (95% CI: 4.4%–54.6%), despite non-seminoma being associated with an earlier age at onset. In addition there is a notable difference in the non-seminoma heritability results from population (49.6%) versus genomic approaches (29.4%), one explanation for which is that the genetic architecture of this sub-type is less dominated by polygenic variation.

## Discussion

In this study we present results from both genomic and population-based techniques, and estimate the heritability of TGCT to be in a consistent range of 37%–49%. The higher estimate from the population-based approach is a logical outcome, given that the pedigree data includes the contribution of all causal variants, whereas the genomic approach can only account for the variation explained by variants in linkage disequilibrium (LD) with genotyped SNPs. This difference is often referred to as missing heritability and underlines the imperfect LD between genotyped SNPs and causal variants^22^. In addition rare variants, indels and structural alterations, which all have potential to contribute to the heritable risk of cancer, are not generally well-captured by GWAS.

Quantification of heritability for TGCT allows the high familial relative risk (RR) of this cancer to be partitioned into inherited and environmental components. On the basis of prevalence of 0.005 for TGCT our estimates of heritability translate to a sibling RR of between 3.8 and 5.4. Comparing these estimates to epidemiological studies, which report an overall sibling RR of ~8, suggests that 48%–68% of the excess sibling TGCT risk can be readily ascribed to inherited genetic factors. Importantly, our heritability values represent only the additive genetic variance (*i.e.* narrow sense heritability), not including non-additive effects such as gene-gene or gene-environment interactions. Hence the total proportion of the familial risk attributable to genetic factors may in fact be greater. A notable feature of TGCT is the differing RR factors observed for different male relatives, with the high RR (~8) for brothers of cases contrasted by a lower ~4-fold increase in risk for father-son relationships^8^. Amongst other factors, this pattern has been attributed to a possible recessive mode of inheritance. An alternative hypothesis is that the majority of excess sibling risk is due to shared early-life environmental exposures; our data would fit this model given total heritable factors are estimated to account for a RR of ~4. Clearly multiple complex factors are likely to influence TGCT aetiology, however the importance of early-life environmental factors is supported by the observation that sibling RR depends on the age difference between brothers, with a RR = 10.8 for differences of less 5 years compared to RR = 6.7 for 5 years and greater^23^. This could reflect in utero exposures common to brothers or household factors in childhood.

While non-seminoma heritability was calculated to be lower using our genomic data, no significant difference was observed based on the population analysis. One possible hypothesis from these observations is that total heritable risk is comparable across subtypes; however there is a subtle difference in underlying architecture, with a lower proportion of non-seminoma risk being determined by common polygenic variants. Further analysis with larger sub-group sample sizes is required, to draw definitive conclusions.

We found that the TGCT susceptibility SNPs identified to date through GWAS account for only a moderate proportion (~10%) of TGCT heritability. This is in contrast to the large proportion of the variance explained by the totality of common variants (~38%), and hence provides unequivocal evidence that a significant number of additional TGCT risk SNPs remain still to be discovered. The exact number is unclear and dependant on a multitude of factors. However, assuming the undiscovered SNP set have effect sizes comparable to the most recently identified TGCT risk loci at 16q22.3 (OR = 1.21), 7p22.3 (OR = 1.16), 4q22.2(OR = 1.15) and 3q25 (OR = 1.16), there are likely to exist at least 50 additional risk SNPs. It is more likely that the set of undiscovered SNPs is even larger in number, with a trailing set of effect sizes.

In summary, we report the first ever study to assess TGCT heritability using both genomic and population-based techniques. Our results demonstrate that TGCT is a strongly heritable cancer, with a polygenic model of disease susceptibility. Although environmental factors must play a key role in the development of TGCT risk, our data suggests that genetic factors contribute significantly to disease aetiology. Our findings quantify the total impact of common variation on TGCT risk, suggesting a significant number of additional risk loci remain to be discovered. Full mapping of all common SNPs associated with TGCT may plausibly offer utility in enabling personalised risk profiling for the disease, through construction of polygenic risk scoring (PRS) models, as implemented in other cancer types^24^,^25^,^26^. Overall our findings provide a strong rationale for continuing the search for additional novel risk variants through GWAS-based strategies.

## Methods and Materials

### Population data: Swedish family-cancer database

Our population based heritability calculations were based on the 2015 update of the Swedish family-cancer database that includes all individuals born after 1931 who are residing in Sweden, together with their biological parents, totalling ∼15.7 million individuals^27^. The database was created in 1996 by combining the Swedish cancer registry and the Swedish multigenerational register, and has been updated regularly. In total 9,324 individuals have been diagnosed with TGCT (ICD-7 code 178), of which 5,042 were seminomas (PAD66), 4,071 were non-seminomas (PAD826) and 208 were mixed/indeterminate histology. The distribution of cases by year is shown in supplementary figure 1, with the rapidly rising disease incidence clearly visible. Of the 9,324 cases 5,230 were diagnosed in the last two decades (1993–2012) and the balancing 4,004 from 1958–1992. All ancestors of patients were extracted from the large pedigree file, working iteratively across each generation back to the founding population. This resulted in a pedigree of 39,662 individuals. The entire pedigree consisted of 7,749 families across five generations with a family size ranging from two to 23 individuals. In addition there were 1,399 singleton TGCT cases. The total number of founders was 23,806 and each family contained at least one and up to three cases.

### Population data: Statistical analysis

A generalized linear mixed effect ordinal model with a binary response variable using Markov chain Monte Carlo (MCMC) algorithm (*e.g.* Gibbs sampler) was applied. Calculations and data analysis were performed using R (version 3.12) packages ‘MCMCglmm’, ‘coda’ and ‘kinship2′. The following parameters were used for the MCMCglmm analysis: i) ‘animal’ model as the formula for random effects, ii) ‘ordinal’ option for trait distributions, iii) χ^2^ prior distribution, iv) sampling chain of 1,100,000 rounds, with 100,000 iterations as burn-in and 1 million sampling rounds. From the MCMC simulations every 1,000^th^ sample was drawn, giving a total of 1,000 samples. Fixed effects included in the model were birth year, birth month, sex, country of birth, social economic index and number of offspring. Calculations were also cross-validated using the software package DMU^28^.

### Genomic data: Quality control

This analysis was based on a previously published GWAS of 986 TGCT cases against 4,946 population controls^10^,^13^. Case samples had a prior diagnosis of TGCT and were taken from two studies (1) a UK study of familial testicular cancer and (2) a national collection of TGCT cases treated within the UK. The studies were co-ordinated at the Institute of Cancer Research (ICR) with samples and information obtained with full informed consent and national ethical review board approval (MREC02/06/66 and 06/MRE06/41). Cases of TGCT were genotyped on the Illumina HumanCNV370-Duo bead arrays. Controls were healthy individuals from the 1958 Birth Cohort genotyped on Illumina Infinium 1.2M array as part of the Wellcome Trust Case Control Consortium^10^,^13^. Our analysis was based on 314,861 SNPs successfully genotyped on both arrays. Individuals were excluded on the following criteria: low call rate (<99%), abnormal autosomal heterozygosity or with >10% non-Western European ancestry (based on multi-dimensional scaling). Strict filtering was applied to remove SNPs with (i) minor allele frequency (MAF) <1%, (ii) a call rate of <95% in cases or controls or (iii) MAF 1–5% and a call rate of <99% or (iv) deviation from Hardy-Weinberg equilibrium (*P* < 0.05). Inflation in the test statistics was observed at only modest levels, rendering substantial cryptic population substructure unlikely (genomic inflation factor^29^ (λ) = 1.08, equivalent to the inflation for a study of 1,000 cases/controls of (λ_1000_) = 1.05). Post QC the series provided 283,274 SNP genotypes on 922 cases and 4,842 controls. Quality control filtering was performed using PLINK (v1.07) software^30^.

### Genomic data: Statistical analysis

GCTA was used to quantify TGCT heritability, estimating the heritability explained by: firstly, all SNPs across the autosome, secondly each individual chromosome and thirdly the 19 established TGCT risk SNPs previously identified by GWAS. For each analysis a genetic relationship matrix (GRM) of pairs of samples was used as input for the restricted maximum likelihood (REML) analysis to estimate the heritability explained by the selected set of SNPs. For the first analysis a single GRM was computed for all autosomal SNPs whereas for the second analysis a GRM was computed for each chromosome individually and then fitted simultaneously for all chromosome GRMs. Finally in the third analysis the heritability for each known SNP was estimated for all chromosomes simultaneously using the risk SNP genotype as a covariate. The heritability associated with the SNP was taken to be the difference between the heritability of the chromosome to which it mapped with and without covariate inclusion. To calculate histology specific heritability the first analysis (all autosomal SNPs) was repeated for seminoma and non-seminoma samples only.

As advocated for diseases such as a cancer, the lifetime-risk rather than the prevalence was used to transform the estimated heritability to the liability scale^31^,^32^. The lifetime-risk for TGCT was set at 0.005^33^, which is closely comparable with TGCT prevalence. The analyses were not adjusted for principal components as the inflation factor was modest. An alternative approach to GCTA is PCGC regression, developed to correct for potential bias introduced by GCTA when converting heritability calculated on the observed binary disease phenotype to the unobserved liability scale^34^. To ensure no such bias was introduced in our estimates analyses were repeated using PCGC, in conjunction with the same GRM as input to estimate heritability by regression.

## Additional Information

**How to cite this article**: Litchfield, K. *et al.* Quantifying the heritability of testicular germ cell tumour using both population-based and genomic approaches. *Sci. Rep.* **5**, 13889; doi: 10.1038/srep13889 (2015).

## Supplementary Material

Supplementary Information

## Figures and Tables

**Table 1:** Estimates of the variance explained by individual chromosomes.

Chromosome	Fraction of Variance Explained
1	0.0422 ± 0.0150
2	0.0232 ± 0.0143
3	0.0506 ± 0.0140
4	0.0313 ± 0.0129
5	0.0017 ± 0.0122
6	0.0248 ± 0.0128
7	0.0178 ± 0.0116
8	0.0095 ± 0.0112
9	0.0200 ± 0.0115
10	0.0124 ± 0.0118
11	0.0192 ± 0.0111
12	0.0339 ± 0.0119
13	0.0058 ± 0.0095
14	0.0117 ± 0.0093
15	0.0150 ± 0.0091
16	0.0083 ± 0.0097
17	0.0188 ± 0.0093
18	0.0143 ± 0.0096
19	0.0050 ± 0.0080
20	0.0342 ± 0.0104
21	0.0033 ± 0.0062
22	0.0000 ± 0.0069
Total	0.3736 ± 0.0500

**Table 2:** Estimates of the variance explained by individual TGCT risk SNPs.

SNP	Odds Ratio	Locus	Gene(s)	Fraction of Variance Explained
rs2072499	1.19	1q22	non-coding	0.0030 ± 0.0211
rs3790672	1.20	1q24.1	non-coding	0.0013 ± 0.0211
rs10510452	1.24	3p24.3	*DAZL*	0.0029 ± 0.0197
rs1510272	1.16	3q25	*SSR3/TIPARP*	0.0048 ± 0.0197
rs17021463	1.15	4q22.2	*HPGDS*	0.0035 ± 0.0182
rs2720460	1.24	4q24	*CENPE*	0.0046 ± 0.0181
rs4635969	1.54	5p15	*TERT*	0.0001 ± 0.0171
rs4624820	1.37	5q31	*SPRY4*	0.0017 ± 0.0172
rs3805663	1.25	5q31.1	*CATSPER3/PITX1*	0.0001 ± 0.0172
rs210138	1.50	6p21	*BAK1*	0.0108 ± 0.0178
rs12699477	1.16	7p22.3	*MAD1L1*	0.0049 ± 0.0162
rs7010162	1.22	8q13.3	*PRDM14*	0.0012 ± 0.0157
rs755383	1.37	9p24	*DMRT1*	0.0144 ± 0.0159
rs995030	2.55	12q21	*KITLG*	0.0177 ± 0.0163
rs2900333	1.27	12p13	*ATF7IP*	0.0028 ± 0.0167
rs8046148	1.32	16q12.1	*HEATR3*	0.0044 ± 0.0136
rs4888265	1.20	16q22.3	*RFWD3*	0.0013 ± 0.0136
rs9905704	1.21	17q22	*RAD51C/TEX14*	0.0095 ± 0.0104
rs2839243	1.26	21q22.3	non-coding	0.0033 ± 0.0090
Total				0.0921 ± 0.0735
