# Supplementary material for: Quantifying the heritability of testicular germ cell tumour using both population-based and genomic approaches
Source: Sci Rep. 2015 Sep 9;5:13889. doi: 10.1038/srep13889 (PMC4563562; doi:10.1038/srep13889)

**Quantifying the heritability of testicular germ cell tumour using both population-based and genomic approaches**

Kevin Litchfield^1^, Hauke Thomsen^2^, Jonathan S. Mitchell^1^, Jan Sundquist^3,4^, Richard S Houlston^1^, Kari Hemminki^2,3^, Clare Turnbull^1,5^,

1. Division of Genetics and Epidemiology, The Institute of Cancer Research, London, SW3 6JB, UK
2. German Cancer Research Center (DKFZ), Division of Molecular Genetic Epidemiology, Heidelberg, Germany
3. Center for Primary Health Care Research, Lund University, Malmö, Sweden
4. Stanford Prevention Research Center, Stanford University School of Medicine, Stanford, CA, USA
5. William Harvey research Centre, Queen Mary University London, London

Correspondence to: Clare Turnbull, Division of Genetics and Epidemiology, The Institute of Cancer Research, London, SW3 6JB; Tel: ++44 (0) 208 722 4485; E-mail: [clare.turnbull@icr.ac.uk](mailto:clare.turnbull@icr.ac.uk)

###### SUPPLEMENTARY FIGURES AND TABLES

###### Supplementary Figure 1 – Number of TGCT cases per year in Swedish population dataset

######
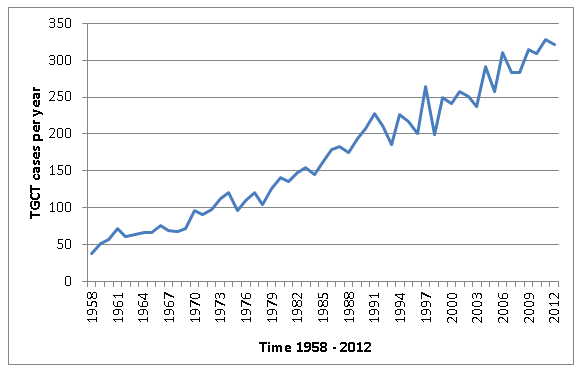

Supplement: Supplementary Information [file srep13889-s1.doc]
